# The complete female mitogenome of *Potomida semirugata* (Lamarck, 1819)

**DOI:** 10.1080/23802359.2024.2378964

**Published:** 2024-07-17

**Authors:** Ana Matos, André Gomes-dos-Santos, Ümit Kebapçı, Mustafa Emre Gürlek, Elsa Froufe, Manuel Lopes-Lima

**Affiliations:** a CIIMAR/CIMAR – Interdisciplinary Centre of Marine and Environmental Research, University of Porto, Matosinhos, Portugal; b Biology Department, Faculty of Science and Arts, Burdur Mehmet Akif Ersoy University, Burdur, Turkey; c Burdur Vocational School of Food Agriculture and Livestock, Mehmet Akif Ersoy University, Burdur, Turkey; d CIBIO, Centro de Investigação em Biodiversidade e Recursos Genéticos, InBIO Laboratório Associado, Universidade do Porto, Vairão, Portugal; e BIOPOLIS Program in Genomics, Biodiversity and Land Planning, CIBIO, Vairão, Portugal

**Keywords:** Freshwater mussels, phylogeny, doubly uniparental inheritance, F-type mitochondrial DNA

## Abstract

Freshwater mussels perform important ecological functions in ecosystems, such as water filtration and energy cycling. Unlike marine bivalves, freshwater mussels have unique characteristics including internal fertilization and parental care. Some freshwater mussels are facing a high risk of extinction due to several factors such as climate change and habitat loss. *Potomida semirugata* (Lamarck, 1819) is one of the freshwater mussel species with a high risk of extinction and listed as Endangered in the Red List of the International Union for Conservation of Nature. Here, we present the first F-type mitogenome sequence of *P. semirugata*. The genome was sequenced on an Illumina high-throughput platform from a *P. semirugata* specimen collected from the Tersakan River (Turkey). The 16,093 bp mitochondrial genome sequence contains 13 protein-coding genes, 22 transfer RNAs, and two ribosomal RNAs. Phylogenetic analysis placed *P. semirugata* in the Lamprotulini clade with *Potomida littoralis*, as expected. *Potomida semirugata* is a poorly studied species and the genomic resource provided here will contribute to a better understanding of its biological characterization.

## Introduction

Freshwater bivalves of the Unionida order, also known as freshwater mussels, belong to an old (>200 Mya) and speciose monophyletic group of bivalves that live exclusively in freshwater (Strayer 2008; Haag 2012; Lopes-Lima et al. 2017). Unlike marine bivalves, freshwater mussels exhibit internal fertilisation and parental care. Their specialised larvae, known as glochidia, require attachment to a host, usually a freshwater fish, for dispersion and nutrition until they metamorphose into juveniles and drop into the substrate (Graf and Cummings 2006; Modesto et al. 2018). In addition, some freshwater mussels have a unique mitochondrial inheritance process, called Doubly Uniparental Inheritance (DUI) (Hoeh et al. 1996). In DUI, males inherit mitochondrial DNA from their mothers (F-type) (present in somatic tissue) and mitochondrial DNA from their fathers (M-type) (present in gonadal tissue) while the female species only inherits the F-type mitochondrial DNA from their mothers (Hoeh et al. 1996). Moreover, the female and male mitochondrial genomes are highly divergent (Froufe, Gan, et al. 2016) with different gene order rearrangements (Huang et al. 2013).

Freshwater mussels play important ecological roles, including water filtration, energy and nutrient cycling, and bioturbation or sediment mixing (Vaughn 2018). They also provide valuable ecosystem services to humans, including increasing water transparency, serving as a source of protein, and providing pearls and shell materials (Strayer 2017). However, they have experienced significant global population declines over the past few centuries, and many have become imperiled (Lopes-Lima et al. 2014, 2018; Lopes-Lima, Riccardi, et al. 2021). As a result, they are now considered one of the most threatened groups of organisms worldwide (Lydeard et al. 2004; Ferreira-Rodríguez et al. 2019). Previously thought to be a monotypic genus consisting only of *Potomida littoralis* (Cuvier, 1798), recent research have shown that the circum-Mediterranean *Potomida* Swainson, 1840 comprises three species with disjunct ranges (Froufe, Prié, et al. 2016). *Potomida littoralis* (Cuvier, 1798) is found in the western Mediterranean area, while *Potomida acarnanica* (Kobelt, 1879) has an isolated distribution area in western Greece. *Potomida semirugata* has an eastern Mediterranean range extending from the Tersakan River basin in the west to the Ceyhan and Asi (=Orontes) basins in the east, along southern Anatolia, the coastal rivers of the Levant, and the Jordan River basin (Lopes-Lima, Gürlek, et al. 2021). Other than that, very little is known about the ecology and habitat requirements of this species. The species is at high risk of extinction and is listed as Endangered in the IUCN Red List (as *P. littoralis*) due to habitat fragmentation and loss (Lopes-Lima et al. 2014), particularly in the south of its range where the species is largely restricted, and coastal streams are either lost or severely disturbed. Given its high conservation profile and limited understanding of its biology, *P. semirugata* must receive scientific attention.

## Materials and methods

**Figure 1.**
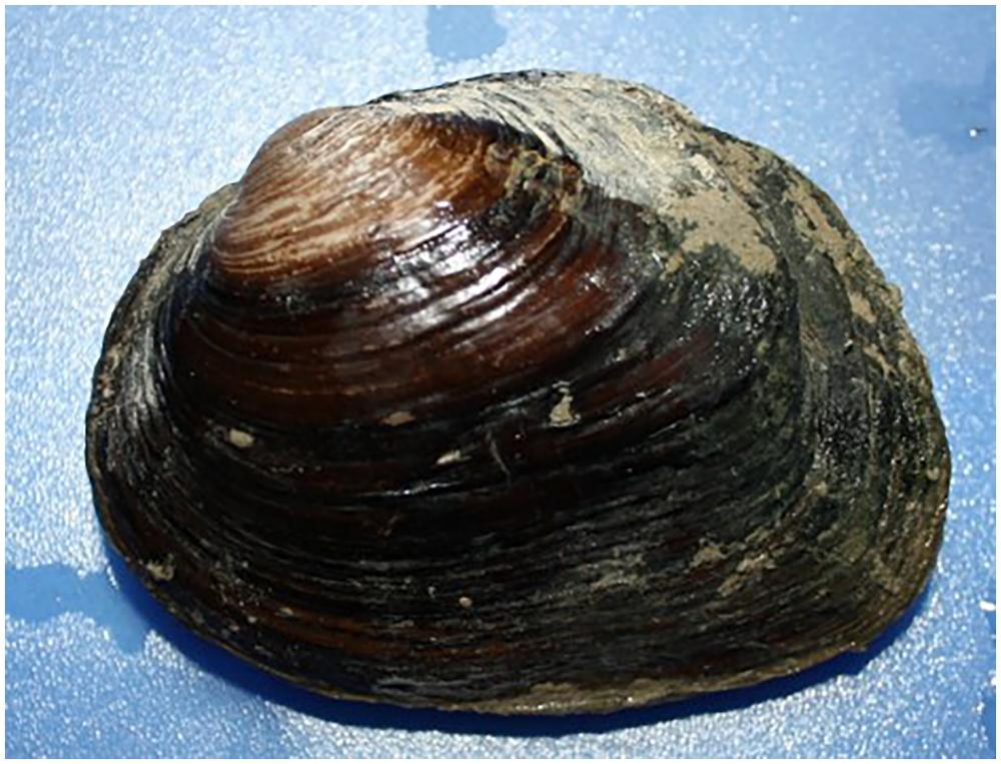
Species image reference of *Potomida semirugata* (photograph by Manuel Lopes-Lima).

A *P. semirugata* specimen was collected on 2 September 2015 at Tersakan River, Muğla, Turkey (36.737379, 28.808708) by Manuel Lopes-Lima (Figure 1). The voucher specimen has been deposited in the Kebapçı collection in the Science and Arts Faculty, Mehmet Akif Ersoy University, Burdur, Turkey (https://www.mehmetakif.edu.tr, Manuel Lopes-Lima, manuelpmlopeslima@gmail.com), with voucher name KC_MAEU BIV2101. Genomic DNA was extracted from the foot tissue of *P. semirugata*, using a high-salt protocol (Aljanabi and Martinez 1997). After extraction, DNA was sent to the Deakin Genomics Centre (Melbourne, Australia) for Illumina Paired-End (PE) library construction (2 × 150 bp) and whole genome sequencing in a MiSeq Illumina platform. The complete F-type mitogenome was assembled using the default parameters, with NOVOPlasty (v.4.2) (Dierckxsens et al. 2017) providing a COI sequence from the same species (KU946889) and annotated with MITOS2 web server (Bernt et al. 2013), respectively. The coverage plot of the mitogenome was obtained using bam2plot (https://github.com/willros/bam2plot) (Supplementary Figure 1). Briefly, the PE reads were mapped to the final mitogenome assembly with Burrows–Wheeler Aligner v.0.7.17-r1198 (Li 2013) using BWA-MEM to generate a sam file. Subsequently, the samtools (version 1.9) was used to convert the sam file in a bam file using option ‘view’ (parameters -Sb) and sorted the alignment using the option ‘sort’ (Danecek et al. 2021). The resulting sorted bam file was used as input to bam2plot to generate the plot (default parameters) (Supplementary Figure 1). From GenBank, 17 F-type Gonideinae mitochondrial genomes were downloaded. Two mitogenomes were also retrieved (from *Amblema plicata* and *Margaritifera margaritifera*) from GenBank as the outgroup. The 13 protein-coding genes from these mitogenomes retrieved from GenBank were aligned with MAFFT (version 7.505) (Katoh and Standley 2013). The alignment was trimmed with trimAL (version 1.2) (Capella-Gutiérrez et al. 2009) and concatenated with FasConCAT-G (version 1.05.1) (Kück and Longo 2014). The final alignment had a total length of 11,148 bp. Using IQ-TREE (version 1.6.12) (Nguyen et al. 2015; Kalyaanamoorthy et al. 2017) partition-scheme and best-fit nucleotide substitution models were identified, and maximum-likelihood phylogeny was conducted. The evolutionary models applied were GTR + F + R4 (*ATP6*, *ND3*, *ND4*, *ND5*), TPM3 + F + I + G4 (*ATP8*), TN + F + I + G4 (*COIII*), TN + F + I + G4 (*COII*), TN + F + R4 (*COI*), TPM3 + F + R4 (*Cytb*, *ND1*, *ND2*), HKY + F + I + G4 (*ND4L*), and TPM3 + F + I + G4 (*ND6*).

## Results

**Figure 2.**
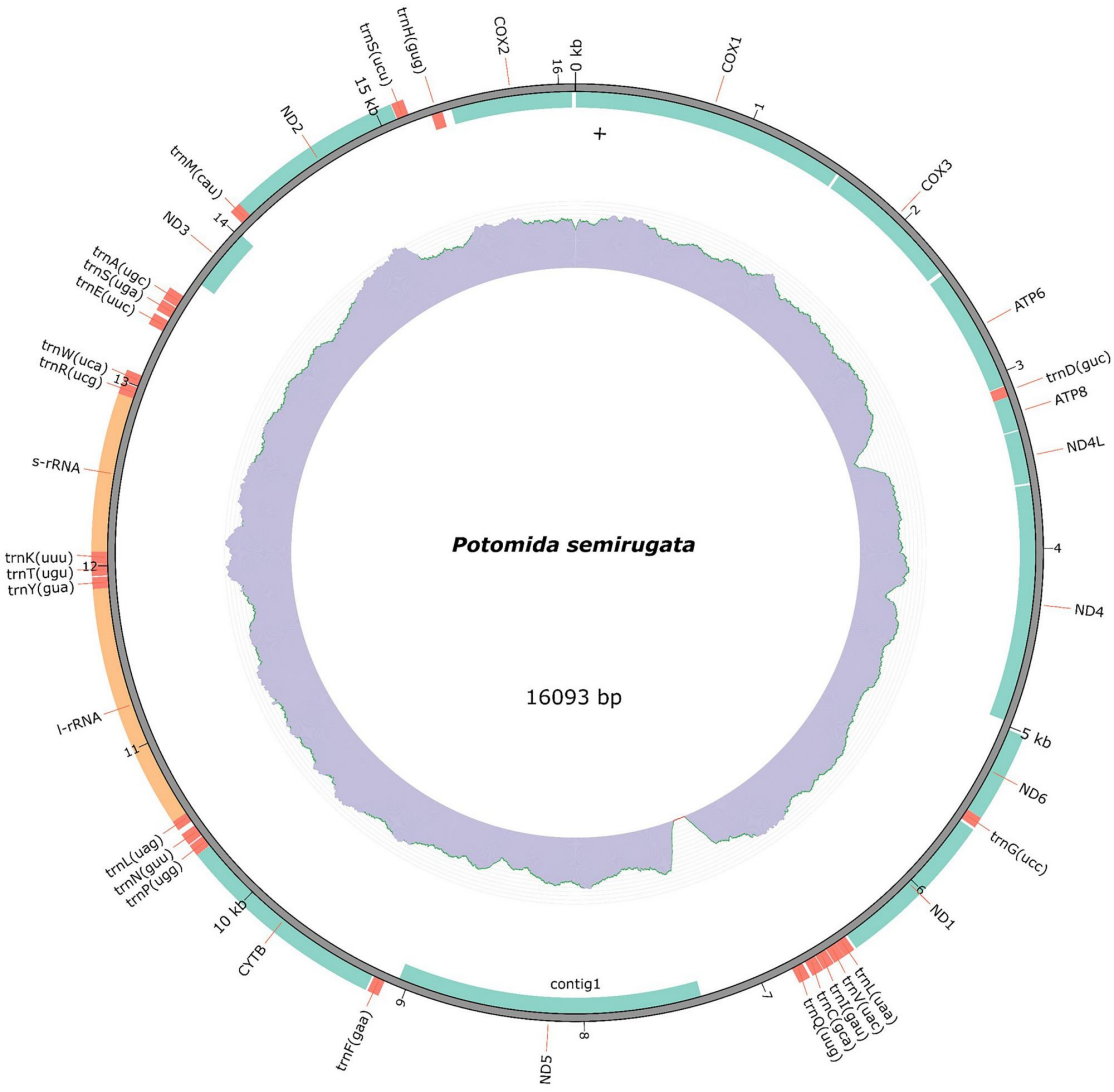
Mitogenome map of *Potomida semirugata*. The plot, created with the annotation model of MITOZ, displays the gene features and their strand positioning on the assembly. PCGs are in green, tRNAs are in red, and rRNAs are in orange. The read depth distribution is displayed in the middle track and GC content in the innermost track.

The F-type mitochondrial genome of *P. semirugata* has a length of 16,093 bp (Figure 2). The coverage plot shows an increased peak between ∼6900 and 7200 bp (Supplementary Figure 1). Several assembly strategies and parameters were tested to validate the integrity of this region. In these attempts, a circular molecule was generated and the peak persisted suggesting the existence of a repetitive motif. Moreover, to validate the integrity of this region, a blast search was conducted against the mtDNA of congeneric species available at NCBI, showing that this region is present with a high percentage of identity and query coverage. This mitogenome has 13 protein-coding genes, 22 transfer RNA (tRNA), and two ribosomal RNA (rRNA). Except for the three *cytochrome c oxidase* subunits (*COX1*, *COX2*, and *COX3*), *ATP synthase F0* subunits* 6* and *8* (*ATP6* and *ATP8*), five *NADH dehydrogenase* subunits (*ND3*, *ND4*, *ND4L*, and *ND5*), and two tRNA (tRNA^Asp^, and tRNA^His^), the other genes are in the complementary strand.

**Figure 3.**
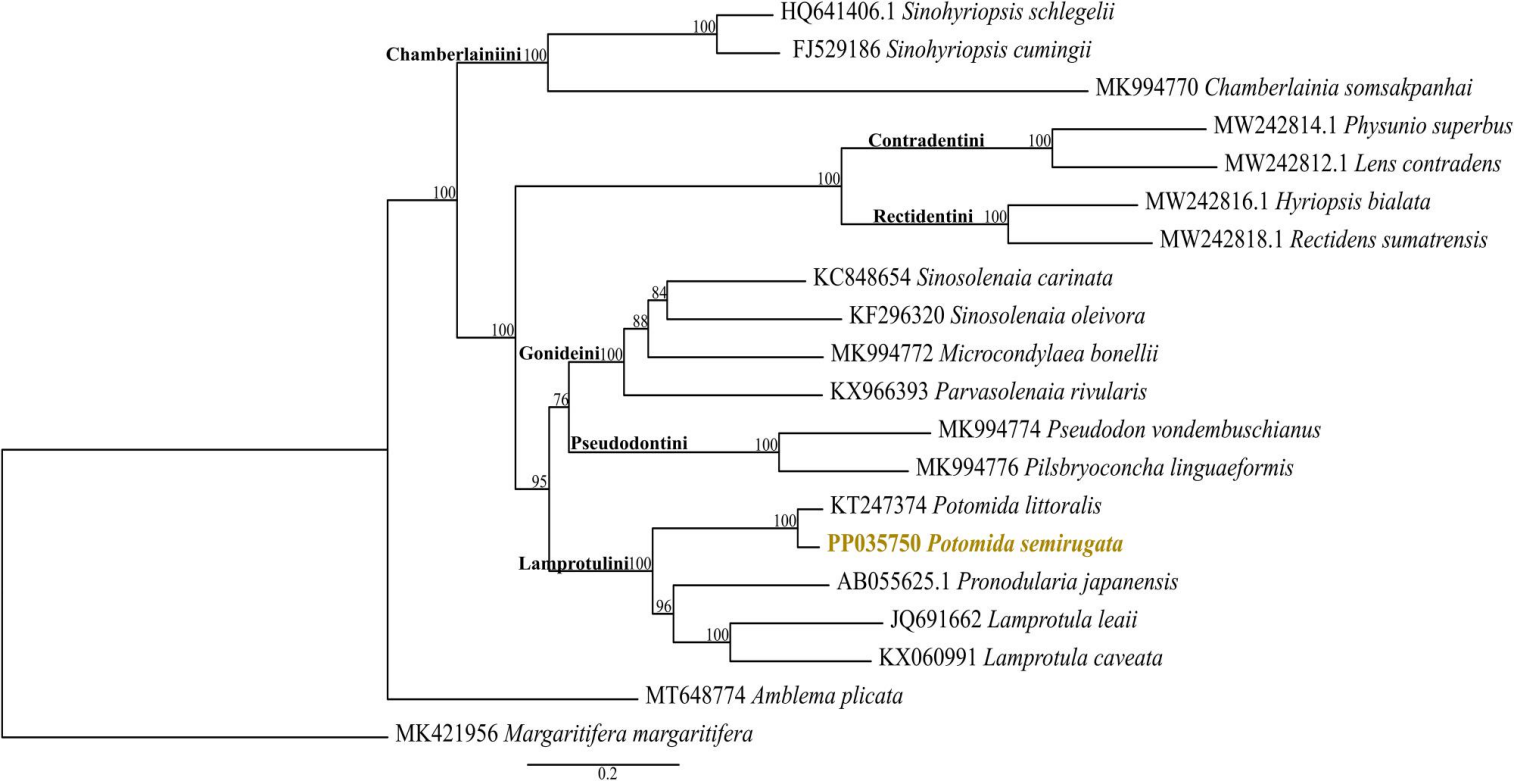
Maximum-likelihood phylogeny with the downloaded mitogenomes (*n* = 19) and with the mitochondrial genome of *Potomida semirugata* (deposited in GenBank with accession number PP035750). The following sequences, retrieved from GenBank, were used: *Sinohyriopsis schlegelii* (HQ641406.1, Sheng et al. 2014), *Sinohyriopsis cumingii* (FJ529186, unpublished), *Chamberlainia somsakpanhai* (MK994770, Froufe et al. 2020), *Physunio superbus* (MW242814.1, Zieritz et al. 2021), *Lens contradens* (MW242812.1, Zieritz et al. 2021), *Hyriopsis bialata* (MW242816.1, Zieritz et al. 2021), *Rectidens sumatrensis* (MW242818.1, Zieritz et al. 2021), *Sinosolenaia carinata* (KC848654, Huang et al. 2013), *Sinosolenaia oleivora* (KF296320, Huang et al. 2015), *Microcondylaea bonellii* (MK994772, Froufe et al. 2020), *Parvasolenaia rivularis* (KX966393, unpublished), *Pseudodon vondembuschianus* (MK994774, Froufe et al. 2020), *Pilsbryoconcha linguaeformis* (MK994776, Froufe et al. 2020), *Potomida littoralis* (KT247374, Froufe, Gan, et al. 2016), *Pronodularia japanensis* (AB055625.1, unpublished), *Lamprotula leaii* (JQ691662, unpublished), *Lamprotula caveata* (KX060991, unpublished), *Amblema plicata* (MT648774, Teiga-Teixeira et al. 2020), and *Margaritifera margaritifera* (MK421956, Gomes-dos-Santos et al. 2019).

The mitogenomes represented in the phylogenetic analysis are grouped according to the described tribes within the family (Figure 3) (Pfeiffer et al. 2019). In the phylogenetic inference, *P. semirugata* is recovered monophyletic with *Potomida littoralis*, in the Lamprotulini tribe (Figure 3). These results are also congruent with the phylogenetic inference provided by Froufe, Gan, et al. (2016).

## Discussion and conclusions

To date (26 December 2023), only two mitogenomes from the *Potomida* genus were publicly available (Froufe, Gan, et al. 2016; Froufe, Prié, et al. 2016), i.e. the female and male mitochondrial genomes of *Potomida littoralis* (Froufe, Gan, et al. 2016; Froufe, Prié, et al. 2016). Here, we provide the first F-type mitogenome of *P. semirugata* whose length (16,093 bp) is within the range of the *P. littoralis* mitochondrial genome already published (Froufe, Gan, et al. 2016). The available number of M-type mitochondrial DNA sequences is less than F-type mitochondrial DNA sequences. However, phylogenetic analysis with both male and female mitochondrial DNA sequences has revealed a high divergence between these sequences being divided into two main clades (one with F-type and the other with M-type sequences) (Froufe, Gan, et al. 2016). Given this divergence, the phylogenetic reconstruction here demonstrated has only F-type mitochondrial sequences. The F-type mitogenome of *P. semirugata* grouped with the F-type mitochondrial sequence of *P. littoralis* in the Lamprotulini clade, as expected. *P. semirugata* is a poorly studied species with a high risk of extinction and is listed as Endangered on the IUCN Red List (Lopes-Lima et al. 2014). Future analyses of mitochondrial genomes may clarify phylogeographic and population genetic patterns, aiding in prioritizing highly genetically diverse or unique populations of *P. semirugata* for conservation.

## Supplementary Material

Supplementary_Material_Psemirugata_180524.docx

## Data Availability

The genome sequence data that support the findings of this study are openly available in GenBank of NCBI at https://www.ncbi.nlm.nih.gov under the accession number PP035750. The associated BioProject, SRA, and Bio-Sample numbers are PRJNA1056155, SRR27332414, and SAMN39090757, respectively.
