# Supplementary material for: The complete female mitogenome of Potomida semirugata (Lamarck, 1819)
Source: Mitochondrial DNA B Resour. 2024 Jul 17;9(7):892–6. doi: 10.1080/23802359.2024.2378964 (PMC11257011; doi:10.1080/23802359.2024.2378964)
Supplement: Supplementary_Material_Psemirugata_180524.docx [file TMDN_A_2378964_SM4452.docx]

**Supplementary Material**

The complete female mitogenome of *Potomida semirugata* (Lamarck, 1819)

Ana Matos^1^, André Gomes-dos-Santos^1^, Ümit Kebapçı^2^, Mustafa Emre Gürlek^3^, Elsa Froufe^1^, Manuel Lopes-Lima^4,5,*^

^1^CIIMAR/CIMAR - Interdisciplinary Centre of Marine and Environmental Research, University of Porto, Matosinhos, Portugal

^2^Biology Department, Faculty of Science and Arts, Burdur Mehmet Akif Ersoy University, Burdur, Türkiye

^3^Burdur Vocational School of Food Agriculture and Livestock, Mehmet Akif Ersoy University, 15100 Burdur, Türkiye

^4^CIBIO, Centro de Investigação em Biodiversidade e Recursos Genéticos, InBIO Laboratório Associado, Campus de Vairão, Universidade do Porto, 4485-661 Vairão, Portugal

^5^BIOPOLIS Program in Genomics, Biodiversity and Land Planning, CIBIO, Campus de Vairão, 4485-661 Vairão, Portugal

* **Corresponding author:** Manuel Lopes-Lima - [manuelpmlopeslima@gmail.com](mailto:manuelpmlopeslima@gmail.com), CIBIO, Centro de Investigação em Biodiversidade e Recursos Genéticos (CIBIO), InBIO Laboratório Associado, Campus de Vairão, Universidade do Porto, 4485-661 Vairão, Portugal and BIOPOLIS Program in Genomics, Biodiversity and Land Planning, CIBIO, Campus de Vairão, 4485-661 Vairão, Portugal.


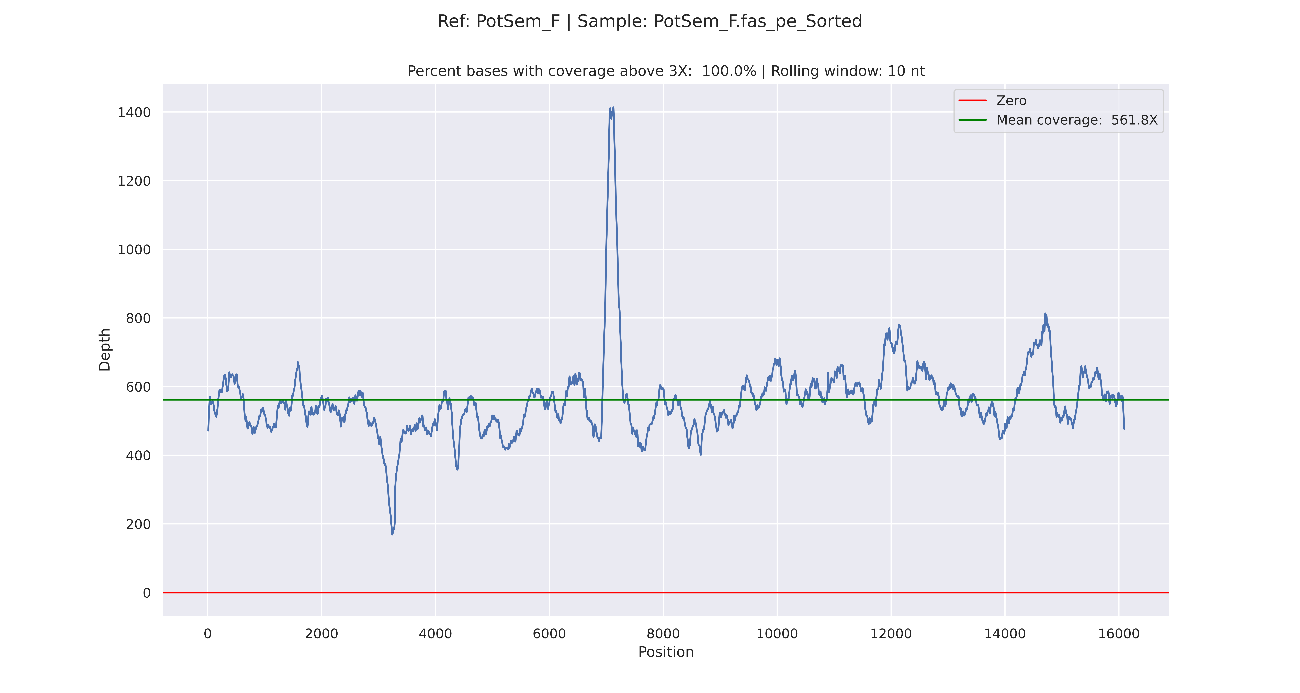


Supplementary Figure 1 - Read coverage plot of *Potomida semirugata* mitogenome.

Supplementary Information 1 – F-ORF from *Potomida semirugata* mitogenome (accession number PP035750).

>F-orf

CTAAGCTTCGGTGATGTCTGTTTGGGCTTTGCTTGGGATTACAGCGTGATTTCCTGTGGTTGTTTGGTTGGTAAGTGTTGGCGGACTAGGTATTGGTGGATCAATGGCTGATACTATTAAGTTGACGGGTAGACCGTGGTATAAAGCTGTGCAGATAAGAATAAGACAGAGTCCCAATAGGATTAAGAGTTTAAGGCTAGCGATGAATTTTTGGAT
